# Assessing water-quality changes in US rivers at multiple geographic scales using results from probabilistic and targeted monitoring

**DOI:** 10.1007/s10661-019-7481-5

**Published:** 2019-05-04

**Authors:** Lori A. Sprague, Richard M. Mitchell, Amina I. Pollard, James A. Falcone

**Affiliations:** 1 National Water Quality Program, US Geological Survey, Denver, CO USA; 2 Office of Water, US Environmental Protection Agency, Washington, DC USA; 3 National Water Quality Program, US Geological Survey, Reston, VA USA

**Keywords:** Rivers, Monitoring, Trends, Chloride

## Abstract

Two commonly used approaches for water quality monitoring are probabilistic and targeted. In a probabilistic approach like the US Environmental Protection Agency’s National Rivers and Streams Assessment, monitoring sites are selected using a statistically representative approach. In a targeted approach like that used by many monitoring organizations, monitoring sites are chosen individually to answer specific questions. One important goal of both approaches is documenting long-term changes in water quality. Here, we compare chloride change results in US rivers and streams between the early 2000s and early 2010s from both approaches. The probabilistic approach provided an unbiased representation of change in all US rivers and streams, but was designed to measure low-streamflow conditions within a spring/summer index period during periodic survey years. The targeted approach was focused on larger, more developed watersheds but samples were collected frequently throughout the assessment period in different seasons and streamflows. The probabilistic results showed a small decrease in chloride concentrations in rivers and streams with the lowest concentrations, but no consistent increase or decrease in the remainder. The increased granularity of the targeted results showed that there was, in fact, a mix of changes occurring, with increases at 132 sites, decreases at 112 sites, and relatively stable conditions at 55 sites. The combined results suggest that chloride is not responding to a widespread, common driver across the USA and that management of chloride would be most effective when targeted regionally or locally.

## Introduction

Maintaining the quality of US rivers and streams is critical for the support of drinking water supplies, aquatic habitat, irrigation, and recreation. Monitoring river and stream quality, interpreting this information, and communicating it to decision makers is the responsibility of many federal, state, tribal, and local water organizations across the USA—from the local level, where on-the-ground management decisions are made, to the federal level, where regional and national policies are set. While each of these stream monitoring efforts may seem similar on the surface, they often are designed with fundamentally different purposes in mind. Each approach answers important questions in their own right, but they can also be complementary. Data from these individual monitoring efforts often can be aggregated to address broader questions, adding value beyond the original goals of the data collection.

In recent years, chloride and salinity pollution in freshwater ecosystems has become a growing concern throughout many regions of the USA (Corsi et al. 2015; Kaushal et al. 2005). Recent studies have shown that increased chloride concentrations can lead to degradation of freshwater biological communities, as well deleterious impacts to drinking water systems (Nelson et al. 2009; Duan and Kaushal 2015; Stets et al. 2018). Understanding how chloride concentrations change at different spatial scales can be valuable information for managers as they develop strategies to protect and improve water quality at a local, regional, and national scale. Utilizing data from multiple monitoring efforts that sample at different spatial scales and temporal frequencies can help to identify the extent of water quality issues in streams and rivers—for example, are the issues limited to a single season, are they limited to a single part of the country, or are they widespread in time and space? Understanding the answers to these questions can help pinpoint the likely causes of the issues and in turn help target management to appropriate seasons and geographic scales.

Two commonly used approaches for monitoring water quality concentrations in rivers and streams of the USA are probabilistic and targeted. In a probabilistic approach, monitoring sites are selected using a statistically representative approach to provide an unbiased estimate of water quality across a population of water bodies. One example of a national-scale probabilistic approach is the National Rivers and Streams Assessment (NRSA), a collaborative program between the US Environmental Protection Agency (USEPA), states, and tribes designed to assess the quality of US rivers and streams using a statistical survey approach (US Environmental Protection Agency 2016). The NRSA monitoring program is intended to provide a national and regional perspective on the condition of flowing water resources. The probabilistic approach used in NRSA relies on a single sample collected across a large number of sites during a spring/summer low streamflow index period, during which the aquatic indicators of interest are generally stable.

Conversely, in a targeted approach, monitoring sites are chosen to answer specific strategic questions that often require more temporally intensive sampling. The targeted monitoring typically is intended to provide detailed understanding of how, when, and why water quality is varying in specific environmental settings, under specific hydrologic conditions, or in response to specific human activities. In this study, we focus on targeted monitoring suitable for determining long-term trends in water chemistry. These targeted approaches rely on a smaller number of sites sampled more frequently and over longer periods of time than is typical of probabilistic approaches, encompassing different seasons and climatic conditions, often over many years. One example of this type of national-scale targeted approach is the US Geological Survey’s (USGS) National Water Quality Assessment (NAWQA) Project, initiated in 1991 to determine the natural and human factors that affect the quality of US rivers and streams in the short and long term (Gilliom et al. 1995). Many other monitoring organizations throughout the USA also employ targeted monitoring.

One important goal of both types of monitoring approaches is documenting long-term changes in water quality and aquatic communities in response to changes in climate, population, land use, water use, management actions, and other factors. The objective of this paper is to examine and compare the use of probabilistic and targeted monitoring data for change analysis at different geographical scales, using the change in chloride concentrations in US streams between the early 2000s and the early 2010s as an illustration.

## Methods

### Probabilistic approach

#### Site selection and sampling

NRSA utilizes a randomized, unequally weighted probability survey approach to select sites that are sampled during a spring/summer index period for each iteration of the survey. This approach allows for an unbiased assessment of the biological and physiochemical condition of the population of perennial rivers and streams across the conterminous USA. The probabilistic approach of NRSA allows for examination of water quality changes over time, evaluation of the stressors affecting aquatic communities, and assessment of the extent of water quality impairment on a regional and national scale. The changes described through probabilistic approaches are occurring at the population level rather than at an individual site level, somewhat analogous to reporting changes in the proportion of the US population that is obese as opposed to weight changes in individuals.

For site selection, the 1:100,000-scale medium-resolution National Hydrography Dataset (NHDPlus version 2) stream network was used (McKay et al. 2012), with a target population consisting of all river and stream reaches that have perennial flowing water. For each survey, a set of new sites was randomly selected from that target population. A subset of sites also was randomly selected from the pool of previously sampled sites to be resampled during the subsequent survey. The change analysis conducted in this study does not focus on just the resampled sites; instead, it includes all sites sampled during each survey to more fully characterize the population of US rivers and streams. However, the inclusion of resampled sites increases the power of the change analysis.

For this paper, we looked at only the stream portion of the target population during the NRSA surveys in 2008–2009 and 2013–2014, and we also included the earlier Wadeable Streams Assessment (WSA) survey in 2000–2004 to extend the time period assessed using the probability approach. To ensure comparability between the three surveys, the stream portion used in this study included only sites on first-through fourth-order reaches (Strahler 1952). The number of first- through fourth-order US stream reaches randomly selected to be sampled in 2000–2004, 2008–2009, and 2013–2014 ranged from 933 to 961. Of these, 356 were resampled between 2000–2004 and 2008–2009 and 520 were resampled between 2008–2009 and 2013–2014.

Each of the three surveys used a standardized sampling method that did not change over time. A 4-L grab sample was collected at each stream during low-streamflow conditions within a spring/summer index period (June 1 through September 30). Sample collection within the stream occurred in an area of flowing water, and usually at the midpoint of the sample reach. All samples were shipped on ice to the analytical laboratory for analysis of chloride and other chemical constituents, usually overnight, with samples arriving within 24 to 48 h after collection (US Environmental Protection Agency 2012, 2013).

### Change analysis

Population estimates for each survey were calculated using a weighted Horvitz-Thompson estimation, with a local mean variance estimator to calculate confidence intervals around the population estimate from each survey (Lohr 1999; Stevens and Olsen 2003). Population estimates were calculated using the spsurvey package in R (R Core Team 2016). To assess the significance of changes between two survey population estimates, a percent change estimate was calculated from each paired combination of the three surveys (i.e., 2000–2004 versus 2008–2009, 2000–2004 versus 2013–2014; and 2008–2009 versus 2013–2014). For a significant change to have occurred between two surveys, the confidence limits for a given change estimate must not have included zero. In addition to assessing change between each survey, an assessment of changes over time was accomplished by comparing the individual cumulative frequency distributions of each survey, both at a national level and within each of the nine aggregate level III ecoregions used by the NRSA program (Omernik 1987).

### Targeted approach

#### Data compilation

Chloride concentration data were compiled from ambient monitoring data that were readily accessible from 277 federal, state, tribal, regional, and local government agencies and nongovernmental organizations. The primary sources of data were the USGS National Water Information System (NWIS) database, the USEPA Water Quality Exchange STOrage and RETrieval (STORET) database, and additional repositories for monitoring data that are not included in STORET (Oelsner et al. 2017). To identify sites suitable for trend analysis between 2002 and 2012, the data were screened to ensure adequate coverage over the full trend period, during each season, and across a range of streamflows, using the following criteria: (1) data were available in either 2002 or 2003 and in either 2011 or 2012; (2) the first 2 years and last 2 years and 70% of years overall in the trend period had at least quarterly samples; (3) at least 14% of the samples in the trend period were high-flow samples—that is, samples collected above the 85th percentile of all historical daily streamflows in the month of a given sample’s collection; (4) no more than 50% of the data set was censored (reported as below the laboratory reporting limit); and (5) the monitoring site was paired with a co-located or nearby streamgage with daily streamflow data available during the entire trend period. A water quality site was only paired with a streamgage site when the respective watershed areas were less than 10% different and there were no intervening influences between the sites. A total of 367 multi-agency targeted trend sites nationwide had chloride data that met these criteria. More detail on data compilation and harmonization, streamgage pairing and the rationale for each screening criterion is provided in Oelsner et al. (2017).

Selected watershed characteristics for the multi-agency-targeted trend sites were taken from Falcone (2017) and Bock et al. (2018), based on watershed boundaries in Falcone et al. (2017). These characteristics included the percent of each watershed in urban land use, based on the National Land Cover Dataset (NLCD) 2011 (Homer et al. 2015), and annual road salt use in each watershed, based on USGS mineral commodity summaries (US Geological Survey 2018). Changes in annual road salt use between 2002 and 2012 were determined by first calculating the Theil-Sen slope through the annual road salt time series using the R package zyp (R Core Team 2016) and then determining the percent change between the 2002 and 2012 points on the slope.

### Trend analysis

The Weighted Regressions on Time, Discharge, and Seasons (WRTDS) model (Hirsch et al. 2010) was used to evaluate trends in chloride concentration between 2002 and 2012 at the multi-agency targeted trend sites. The WRTDS model was implemented using the R package EGRETci 1.0.4 (Hirsch and De Cicco 2015; R Core Team 2016). Because chloride concentrations in streams and rivers can be strongly influenced by variations in streamflow, flow-normalized concentrations were estimated in WRTDS. The flow-normalization process removes the variation in concentration resulting from random or systematic streamflow variations, and thus represents long-term changes in chloride concentration caused by other factors on the landscape (Hirsch et al. 2010). Trends in annual mean flow-normalized concentration were calculated as the difference between annual mean flow-normalized concentration in the start and end year of the trend period.

The 90% confidence intervals and associated likelihood statistic of the trend were determined through a block bootstrap approach based on a time interval of 100 days to avoid oversampling any of the more densely sampled periods during the trend period of record and to broadly maintain samples from individual high or low streamflow events (Hirsch et al. 2015). A trend was “likely up” or “likely down” when the likelihood statistic was between 0.85 and 1.0—in other words, the chance of the trend occurring in the specified direction was at least 85 out of 100. A trend was “somewhat likely up” or “somewhat likely down” when the likelihood statistic was between 0.7 and 0.85. A trend was “about as likely as not” when the likelihood statistic was less than 0.7 (Oelsner et al. 2017).

Diagnostic plots for each model were examined for normality and homoscedasticity of the residuals and a reasonable relationship between observed and estimated values. Of the 367 sites that passed the initial screens and were included in the WRTDS modeling, 299 sites were retained for subsequent analyses after checking model diagnostics. These final sites included data from 30 different monitoring organizations. The final trend results are available in a digital online data set (De Cicco et al. 2017). More detail on model specification and checking is available in Oelsner et al. (2017).

## Results and discussion

### National-scale changes

**Fig. 1:**
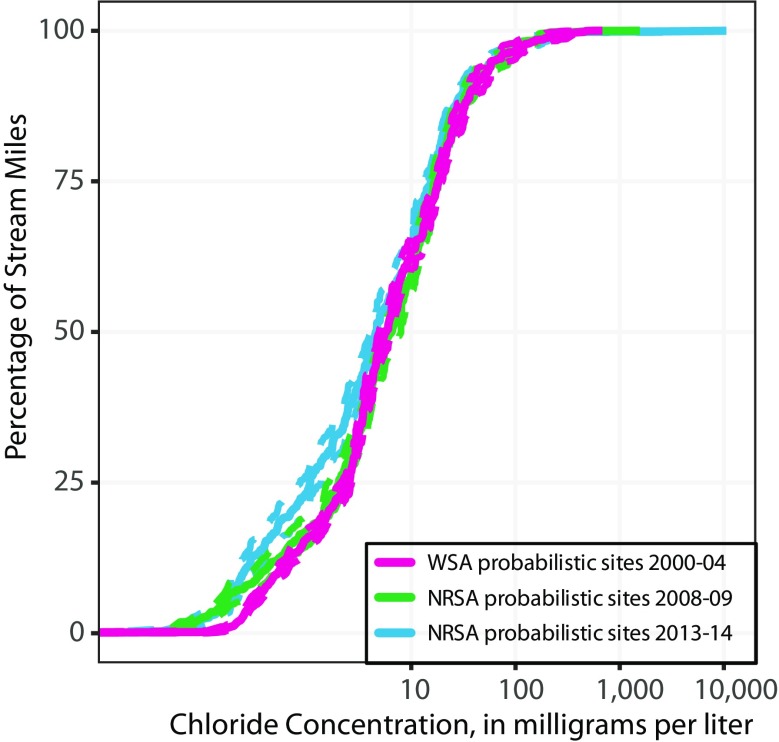
Cumulative frequency distributions of chloride concentrations in the population of rivers and streams in the conterminous USA during the 2000–2004 Wadeable Streams Assessment (WSA) and 2008–2009 and 2013–2014 National Rivers and Stream Assessment (NRSA) probabilistic surveys. Vertical axis is the cumulative percentage of stream miles with concentrations less than or equal to the corresponding horizontal axis value. Solid lines are weighted population estimates and dashed lines are 95% confidence intervals

The NRSA probabilistic surveys are designed to provide population-level estimates of changes—in this case, the population is all U.S. streams. As an example, Fig. 1 shows population estimates for chloride concentrations in US streams during each NRSA probabilistic survey. About 70% of stream reaches had chloride concentrations below 10 mg/L in all three surveys; overall, most concentrations were below the USEPA chronic aquatic-life criteria of 230 mg/L. About 25% of the stream reaches had modest decreases of about 3 mg/L between the 2000–2004 and 2013–2014 surveys. But across all streams, the probabilistic results showed little change in chloride concentrations at a national scale between the 2000–2004, 2008–2009, and 2013–2014 surveys. Because the large spatial scale of NRSA incorporates changes occurring at many individual sites across the population, widespread changes in one direction, either increasing or decreasing, within the USA would be necessary before the change would be captured in population-level results in NRSA. So, the lack of change at a national scale could be because chloride concentrations changed very little at that scale, or because there was a relatively even mix of increasing, decreasing, and stable concentrations throughout the USA.

**Fig. 2:**
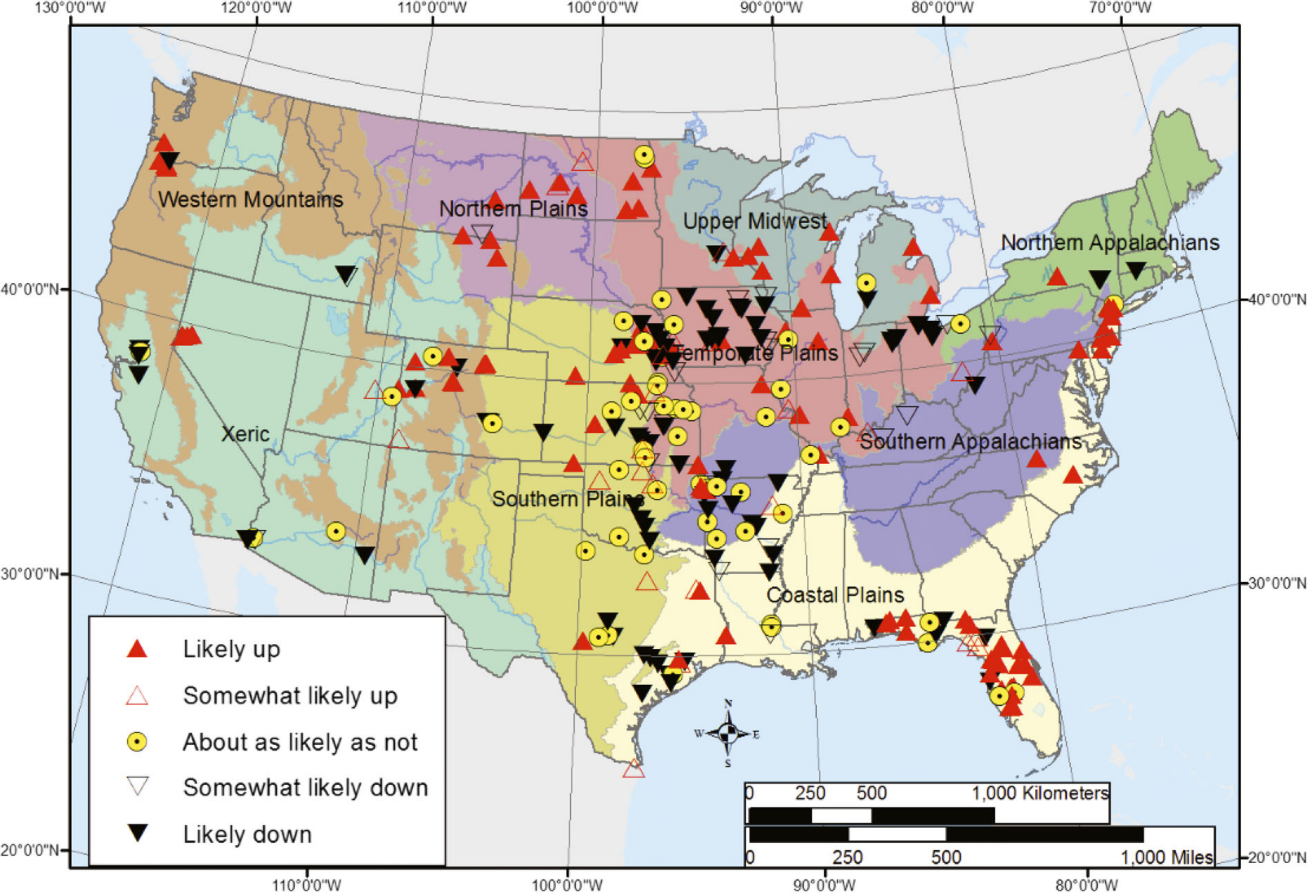
Trends in chloride concentration between 2002 and 2012 at the multi-agency targeted trend sites. Aggregated USEPA Level III ecoregion boundaries also are shown. When the trend is “likely up” or “likely down,” the likelihood value associated with the trend is between 0.85 and 1.0—in other words, the chance of the trend occurring in the specified direction is at least an 85 out of 100. When the trend is “somewhat likely up” or “somewhat likely down,” the likelihood value associated with the trend is between 0.7 and 0.85—in other words, the chance of the trend occurring in the specified direction is between 70 and 85 out of 100. When the trend is “about as likely as not”, the likelihood value associated with the trend is less than 0.7—in other words, the chance of the trend being either upward or downward is less than 70 out of 100

The trend results from the multi-agency-targeted trend sites provide additional insight in the behavior over time in individual streams. For chloride, these results point to the reason for the lack of change at the national scale—chloride concentrations both increased and decreased at a large number of individual multi-agency targeted trend sites during the period contemporaneous with the three probabilistic surveys. Between 2002 and 2012, chloride concentrations were likely or somewhat likely increasing at 132 multi-agency targeted trend sites, likely or somewhat likely decreasing at 112 sites, and relatively stable (trend about as likely as not) at 55 sites (Fig. 2). The fragmented site locations and range in the trend results make it difficult to generalize these results to a unified regional or national pattern, but they provide critical information on the diversity of chloride changes throughout the USA. The more detailed information available at the targeted trend sites also helps to interpret the probabilistic results.

Because of the random and statistically representative nature of probabilistic site selection, the results from the three probabilistic surveys provide an unbiased assessment of changing concentrations in the population of US streams during the spring/summer low streamflow index period, without needing to monitor every stream reach in the USA. The multi-agency-targeted trend sites are numerous and broadly distributed and so can provide some level of national insight. But the multi-agency-targeted sites are not randomly selected and are biased toward larger streams closer to population centers than the probabilistic sites or US stream reaches as a whole (Table 1). Based on an evaluation of the NHDPlus version 2 stream network (McKay et al. 2012), 95% of all US stream reaches are smaller, wadeable first through fourth-order reaches (Strahler 1952); only 5% are larger, higher-order reaches (Table 1). The multi-agency-targeted trend sites included more medium and large rivers and streams, where urban and agricultural development generally is more intense (Lowe and Likens 2005). This may reflect a priority among monitoring organizations to target monitoring in areas with known impacts or with the potential to develop impacts because of ongoing urban or agricultural expansion. Urban and agricultural watersheds previously have been shown to have higher concentrations, more frequent detections, and more complex mixtures of pesticides (Sprague and Nowell 2008; Gilliom 2007; Gilliom et al. 1999) and higher concentrations of nutrients and chloride (Tran et al. 2010; Gardner and Royer 2010; Sprague et al. 2007; Morgan et al. 2007) compared to less developed watersheds. In addition, more money is spent to maintain or improve water quality where greater pollution is occurring. As a result, monitoring organizations may focus more of their monitoring investment in developed watersheds in order to track the return on their management investments. Monitoring organizations also may find it logistically easier to collect samples from streams in these less remote areas closer to their bases of operation, or they may wish to track the accumulated effects of human activities over a large contributing area by monitoring at political boundaries, the outlet of major watersheds, or the inlet to major receiving water bodies. In addition, some organizations expressly monitor at or near drinking water intakes on an ongoing basis to ensure the quality of source water. These intakes tend to be close to population centers (Table 1).

**Table 1:** Selected characteristics of US stream reaches, the Wadeable Streams Assessment (WSA) and National Rivers and Stream Assessment (NRSA) probabilistic sites during three different survey periods, and the multi-agency targeted trend sites. [Stream reach information from NHDPlus Version 2 (McKay et al. 2012), 2010 city locations and populations from ESRI 2017, and drinking water intakes based on data from Price and Maupin 2014. 5 k city = city with 2010 population of 5000 or more]

	Number of stream reaches, sites, or intakes	Percent in lower-order stream reaches^a^	Percent in higher-order stream reaches^b^	Median distance to a 5 k city (km)	Percent within 10 miles of 5 k city
All US stream reaches	2.69 million	95	5	37.5	28
WSA probabilistic sites 2000–2004	954	100	0	33.8	21
NRSA probabilistic sites 2008–2009	961	100	0	25.7	31
NRSA probabilistic sites 2013–2014	933	100	0	26.3	32
Multi-agency-targeted trend sites	299	22	78	13.7	56
Drinking water intakes	10,797	79	21	11.2	61

^a^0–4th Strahler order

^b^Higher than 4th Strahler order

Population estimates from the national NRSA probabilistic surveys rely on the collective characteristics of a large number of surveyed sites and as such, they are best suited for describing widespread, large-scale water quality. National-scale probabilistic programs can provide regional and national context for programs focused on local, targeted questions. For example, total phosphorus concentrations in the population of US rivers and streams increased over the course of the three NRSA surveys, most notably in sites within relatively undisturbed catchments (Stoddard et al. 2016). The widespread nature of these total phosphorus increases suggests a national-scale influence on changing stream quality—possibly an increase in the atmospheric deposition of phosphorus (Stoddard et al. 2016). This type of insight into national-scale influences can help inform policy decisions at the federal level. But the increased granularity of targeted trend results is needed to determine if a uniform federal policy is adequate to protect water resources at a regional or local scale.

### Regional-scale changes

**Fig. 3:**
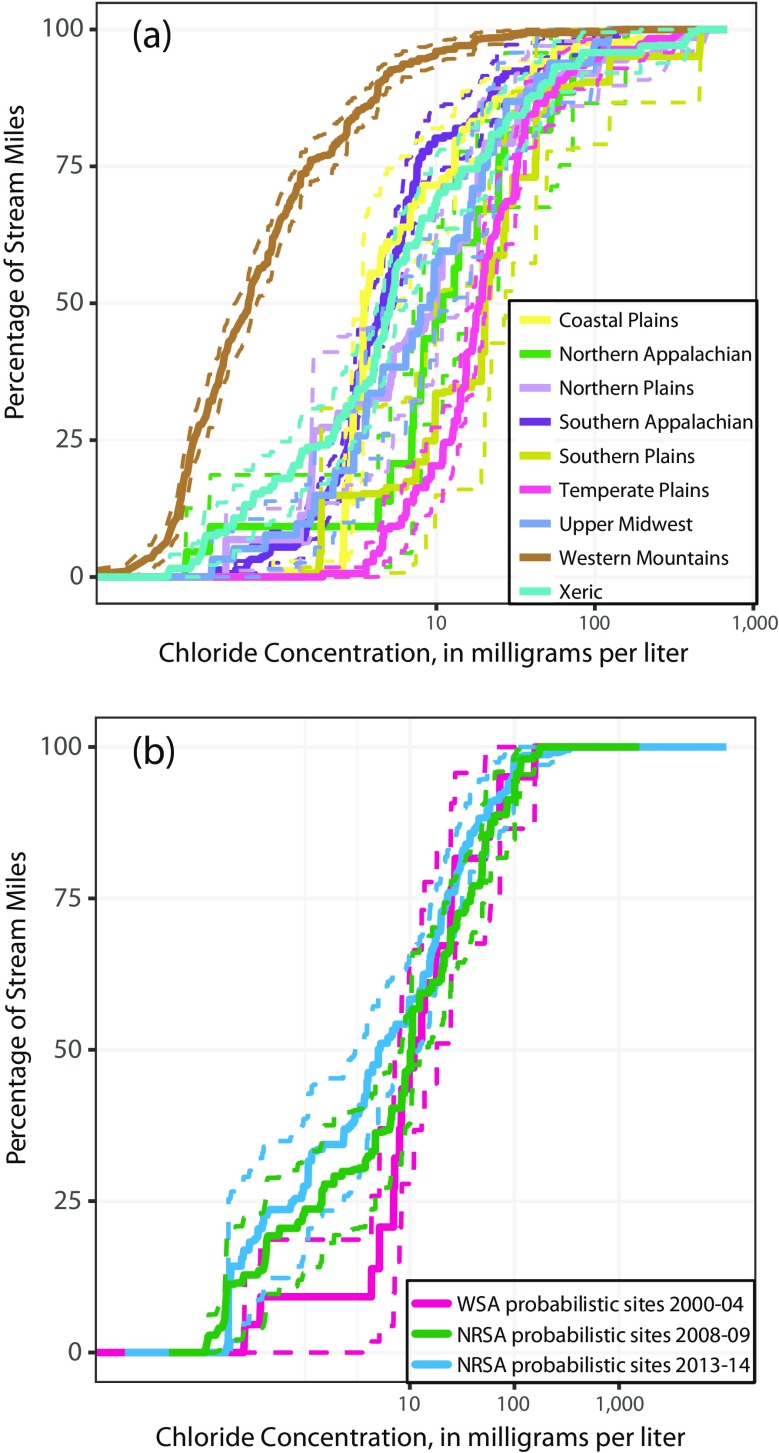
Cumulative frequency distributions of chloride concentrations in the population of streams in **a** aggregate level III ecoregions during the 2000–2004 WSA survey and **b** the Northern Appalachians ecoregions during the Wadeable Streams Assessment (WSA) and the National Rivers and Stream Assessment (NRSA) probabilistic surveys. Vertical axis is the cumulative percentage of stream miles with concentrations less than or equal to the corresponding horizontal axis value. Solid lines are weighted population estimates and dashed lines are 95% confidence intervals

In addition to providing national estimates of water quality, the NRSA probabilistic surveys can be disaggregated to provide regional estimates of water quality. As an example, Fig. 3a shows the chloride concentrations by ecoregion for the 2000–2004 WSA survey, which were similar to the pattern during the other two surveys (data not shown). There were significant differences in ambient chloride concentrations among the nine aggregate level III ecoregions within this single survey period. The ecoregions with the highest concentrations of chloride occurred in the southern and temperate plains, with the lowest concentrations occurring in the western mountains. Further, there were significant changes between survey periods at the ecoregional scale. For example, in the Northern Appalachians, the lower 25th percentile of stream reaches had lower concentrations of chloride during the 2013–2014 NRSA survey compared to the 2000–2004 WSA survey, with chloride concentrations in the 2008–2009 NRSA survey intermediate to the other two surveys (Fig. 3b). There were few multi-agency-targeted trend sites in this ecoregion, and the trend results were mixed (Fig. 2).

These chloride results illustrate two important points about the use of probabilistic and targeted data for change analysis. First, probabilistic change results for geographic regions underrepresented by long-term targeted monitoring provide insight into important changes in regional water quality that are not provided by the targeted results alone. In this case, there were no statistically significant chloride increases in streams at a regional scale. Second, the targeted trend results show that population-scale changes in an ecoregion may not reflect the diversity of changes in individual streams within that ecoregion. In this example, there were both increasing and decreasing chloride concentrations at multi-agency targeted sites within the Northern Appalachians ecoregion.

### Local-scale changes

**Fig. 4:**
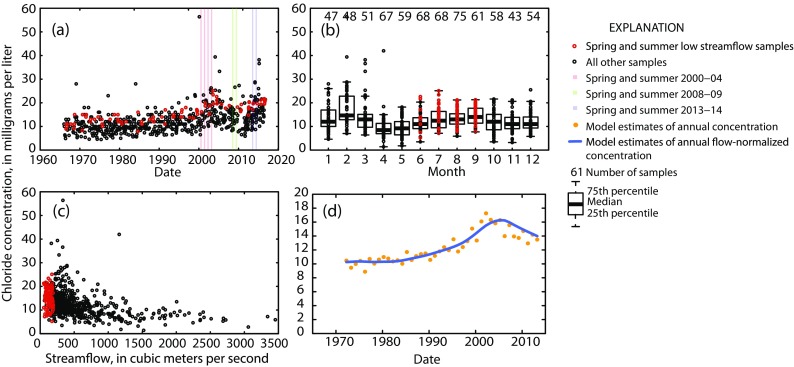
Observed chloride concentrations versus **a** time, **b** month, and **c** streamflow and **d** modeled annual estimated and flow-normalized chloride concentrations versus time in the Connecticut River at Thompsonville, CT, USA (US Geological Survey site 01184000). Samples collected during spring and summer low streamflows—corresponding to the index period of the Wadeable Streams Assessment and the National Rivers and Stream Assessment probabilistic surveys—are shown as open red circles. The index period of the three probabilistic surveys are highlighted in part (**a**). Note this site is a multi-agency targeted-trend site and was not included in the probabilistic surveys

In order to make statistically representative conclusions, the national probabilistic surveys rely on the collective characteristics of a large number of sites. The multi-agency-targeted trend sites can help provide insight into how local water quality has been changing over time and, when monitoring has been long enough, how recent changes compare to decades past. As an example, chloride concentrations in the Connecticut River decreased between 2004 and 2012 (Fig. 4d), which is consistent with the probabilistic results in the Northern Appalachians ecoregion (Fig. 3b). The longer-term picture shows that this decrease followed a more gradual increase between 1972 and 2004 (Fig. 4d). From 1940 to 2000, road salt sales in the USA increased over 1000% (Robinson et al. 2003; Salt Institute 2001). The increases in chloride concentrations in the Connecticut River between 1972 and 2004 generally correspond to this increase in road-salt sales. Road-salt usage in Vermont (comprising 41% of the Connecticut River watershed) decreased by approximately 30% between 1999 and 2009 (Smeltzer et al. 2012), which likely contributed to the decrease in chloride concentrations in the Connecticut River starting in 2004. Smeltzer et al. (2012) also found a lag of about 5 years between the start of the road salt decrease and the start of chloride decreases on the Vermont side of Lake Champlain.

Although all of the factors affecting chloride trends at each individual multi-agency-targeted trend site were not determined as part of this study, previous studies suggest that a number of other factors likely could have been important—changes in inputs from treated wastewater, water-conditioning salts, agricultural fertilizer, oil and gas development, and industrial discharge have been suggested as possible causes of changing chloride concentrations in streams in different parts of the USA (Dailey et al. 2014; Warner et al. 2013; Kelly et al. 2010; Chapra et al. 2009). In many areas, there are likely to be multiple concurrent factors affecting chloride concentrations; the relative influence of each factor will vary locally from watershed to watershed.

Detailed, site-specific results from the multi-agency-targeted trend sites can also be used to evaluate potential impacts to aquatic life or human health at a scale appropriate for local pollution control and water treatment efforts. Elevated chloride concentrations in source water can promote the galvanic corrosion of lead-bearing minerals (Ng and Lin 2016; Willison and Boyer 2012). In the case of the Connecticut River, where there are over 100 drinking water intakes in the watershed in varying proximities to the monitoring location (Price and Maupin 2014), the ratio of chloride concentrations to contemporaneous sulfate concentrations (the chloride-sulfate mass ratio; CSMR) is often above 0.5 (Stets et al. 2018). A CSMR above 0.5 potentially promotes galvanic corrosion of leaded connections in drinking-water distribution systems (Nguyen et al. 2011), which could necessitate additional treatment of source water before distribution. At a subset of the multi-agency-targeted trend sites nationwide, the probability of lead action-level exceedances at 39 drinking-water facilities increased along with the CSMR, indicating a statistical connection between local stream chemistry and corrosion in drinking-water facilities (Stets et al. 2018).

Because chloride concentrations in rivers and streams vary within and among years as the sources of chloride vary seasonally and over time, the continuous long-term monitoring across different seasons and hydrologic conditions at the multi-agency-targeted trend sites also can help provide insight into the factors contributing to the trends, assist with local or watershed adaptive management, and elucidate potential risks to aquatic life and ecosystem health. In rivers located in snow-affected areas of the USA, such as the Connecticut River (Fig. 4), chloride concentrations often are highest in the winter when road-salt usage is highest (Stets et al. 2018; Corsi et al. 2010; Corsi et al. 2015; Dailey et al. 2014). Chloride concentrations also vary in response to changes in streamflow (Corsi et al. 2015; Kelly et al. 2010). For example, in the Connecticut River, the lowest concentrations occur at the highest streamflows when relatively dilute water from storm events enters the river, increasing streamflow and decreasing chloride concentrations (Fig. 4c). The highest concentrations occur at low streamflows, most often in the winter, when road-salt usage is highest and relatively little stormwater is entering the river to dilute the chloride. Because of the inter- and intra-annual variability and streamflow-related variability in chloride concentrations, samples collected consistently across years, seasonally throughout each year, and across the full range of streamflow conditions each year most completely characterize the population of chloride concentrations in a stream or river. The multi-agency-targeted trend sites used in this evaluation were screened to ensure the monitoring data covered a range of seasonal and streamflow conditions consistently across years. The NRSA probabilistic sites, however, were sampled once during each survey period, during low-streamflow conditions in a spring/summer index period (US Environmental Protection Agency 2009). Because of the focus on this spring/summer low-streamflow index period, sampling at the NRSA probabilistic sites underrepresents chloride concentrations at moderate and higher streamflows and during the fall and winter seasons (for example, Fig. 4a–c), but they provide a cost-effective snapshot of low-streamflow conditions across a broad spatial area to assess the effects of potential water quality stressors like chloride on the health of algal, macroinvertebrate, and fish communities that are also sampled during the index period.

## Conclusions

Current funding levels for stream monitoring in the USA are not adequate for frequent monitoring of a large number of statistically representative river and stream sites as conditions continually change within and across years. By combining results from probabilistic and targeted monitoring, the respective strengths of both approaches can be used to bring us closer to that goal. For chloride, the probabilistic results showed a small decrease in concentrations in US rivers and streams with the lowest concentrations, as well as either stable conditions or an indeterminate mix of increases and decreases in the remaining rivers and streams. This finding indicated that there is not a widespread problem of concern with chloride during spring/summer low-streamflow conditions. The increased granularity of the targeted results, however, showed that there was a mix of increases and decreases in individual locations, with an increase in chloride concentrations at 132 targeted sites, a decrease at 112 sites, and relatively stable conditions at 55 sites. These combined results can help inform management at the local level, where on-the-ground management decisions are made, as well as the federal level, where regional and national policies are established (Table 2). The lack of significant change in chloride concentrations in the population of US rivers and streams and the mix of increases and decreases in individual rivers and streams suggest that chloride is not responding to a widespread, common driver across the USA and that management of chloride would be most effective when targeted regionally or locally. These combined results capture the behavior of the population of US rivers and streams during spring/summer low-streamflow conditions (probabilistic results) and supplement that with more detailed site-specific information on continuous long-term changes in rivers and streams within and across years at a more limited number of sites (targeted results) (Table 2). The most complete and robust monitoring approach to support future management of water quality at multiple scales would require changes to stream monitoring in the USA in a way that combines the benefits of both probabilistic and targeted monitoring—sample collection at a large number of statistically representative river and stream sites throughout the year on a continued long-term basis.

**Table 2:** Comparison of the probabilistic and targeted approaches used for change assessments in this study

	Probabilistic	Targeted
Site characteristics	Randomized, unequally weighted sites selected from the population of US rivers and streams	Sites in larger watersheds that are more impacted by human development; often near cities and drinking water intakes
Sample collection approach	Collection during low streamflow conditions in the spring/summer	Collection across the full range of seasons and streamflows
Sample frequency	Once at each site during each of 3 survey periods	At least 4 times per year in each year
Change results	Aggregated, statistically representative change in the population of US rivers and streams. These can sometimes be disaggregated to a regional scale	Individual trend results for each site. These can sometimes be aggregated to a regional or national scale
Scale of relevance	National and regional levels, to help inform regional and national water policy	Local, regional, and sometimes national levels, to help with on-the-ground management decisions
